# Comparative analysis of the clinical efficacy of conservative treatment for adenomyosis using traditional Chinese medicine and Western medicine

**DOI:** 10.1186/s41043-025-00852-z

**Published:** 2025-04-13

**Authors:** Yan Liang, Tianling Xiao, Haiyan Zhang

**Affiliations:** 1 Department of Gynaecology and Obstetrics, Baotou Medical College, Baotou Central Hospital, No. 61 Huancheng Road, Donghe District, Baotou City, 014000 China; 2 Department of Gynaecology and Obstetrics, Baotou Central Hospital, No. 61 Huancheng Road, Donghe District, Baotou City, 014000 China

**Keywords:** Dan’e Fukang Decoction, Dienogest, Goserelin, Mirena, Adenomyosis, Clinical efficacy

## Abstract

**Objective:**

To explore the clinical efficacy of commonly used conservative treatments for adenomyosis using both TCM and Western medicine.

**Methods:**

210 patients with adenomyosis were selected and divided into 3 groups: Group A (Dan’e Fukang), Group B (Dienogest), and Group C (Goserelin + Mirena), with 70 cases in each group. Afterward, indicators were collected for comparison.

**Results:**

Different treatment approaches exhibited varying effects on uterine VAS, and PBAC among the 3 groups (*P* < 0.001). The effects of different treatment approaches on serum levels of estradiol (E2), FSH, and CA125 also differed among the 3 groups (*P* < 0.001). After 3 months of treatment, the incidence of adverse reactions among the 3 groups was significantly different (*P* < 0.001), with further comparison indicating a lower incidence of adverse reactions in Groups A and B than in Group C (*P* < 0.017). Meanwhile, statistically significant differences in the incidence of adverse reactions among the 3 groups were observed again after 6 months of treatment (*P* = 0.004), with further comparison revealing a lower incidence of adverse reactions in Group B than in Group C. Additionally, the comparison of uterine volume (*P* < 0.001) and VAS (*P* < 0.001) among the 3 groups was different after 12 months of treatment, and further comparison revealed that the uterine volume was ranked as Group B > Group C > Group A, while the pelvic pain VAS scores were ranked as Group C > Group B = Group A.

**Conclusion:**

Dan’e Fukang Decoction is markedly effective in alleviating pain; Goserelin + Mirena exhibits significant efficacy in reducing bleeding.

## Introduction

Adenomyosis (AM) is an estrogen-dependent disease primarily caused by the growth of endometrial glands and stroma into the uterine smooth muscle [1]. It has become a common gynecological condition, and recent epidemiological surveys have found that the incidence of this disease is increasing year by year, with the onset in an increasingly younger population [2]. It has been suggested that about 30-35% of women with adenomyosis exhibit symptoms, with most patients aged 32–38 years [3]. Women of childbearing age with AM experience a significant decline in their quality of life, as they are often accompanied by symptoms such as increasingly severe pain, increased bleeding, larger uterine volume, and even infertility [4]. Studies indicate that women with multiple pregnancies, a history of miscarriage, uterine hydrosalpinx, long-term smoking, and irregular menstruation are prone to adenomyosis [5].

The objectives for treating this condition include: starting with improving dysmenorrhea, reducing lesions, and lowering disease recurrence, followed by eliminating dysmenorrhea, completely removing lesions, and preventing disease growth, ultimately achieving the purpose of improving the quality of life. Treatment should comprehensively take into account the age, willingness, extent of lesions, fertility needs, severity of symptoms, and previous treatment history of patients [6]. Treatment approaches include: Expectant management; surgeries that preserve fertility (lesion excision, various modified excision techniques), fertility-sparing surgery (lesion excavation, various modified lesion resection), ovarian-sparing surgery [7] (hysterectomy), radical surgery (total hysterectomy); anti-inflammatory and analgesic medications; short-acting contraceptives; classic gestrinone therapies; GnRH agonists (more common in clinical practice include leuprolide and norethindrone); physical therapies (e.g., transcutaneous electrical acupoint stimulation), traditional Chinese medicine, and minimally invasive interventional treatments (e.g., ultrasound-guided percutaneous microwave ablation, percutaneous focused ultrasound). In this regard, each treatment method has its advantages and disadvantages. The recurrence rate of AM after uterine-sparing surgery is about 5%, and surgery can cause some tissue damage [8], which may lead to inflammatory responses at the surgical site, whereas radical surgery is limited to specific patient populations and can be quite harmful. In the meantime, long-term use of drugs may lead to corresponding side effects, such as masculinization, perimenopausal symptoms, infertility, liver and kidney function impairment, and a high recurrence rate after drug withdrawal. Currently, various interventional surgeries are also emerging, and while Western medicine boasts some advantages in treating adenomyosis, their limitations still remain.

The principle of TCM treatment is “urgent treatment of symptoms, slow treatment of the root cause”. Therefore, TCM has its unique advantages due to its focus on “promoting blood circulation and removing blood stasis” in the treatment of AM [9]. Generally speaking, the uterus is bloated as menstrual blood blocked in the myometrium cannot be dispelled, leading the disease to be prolonged and difficult to treat. Qi stagnation and blood stasis require qi regulation and blood circulation, and the common clinically used traditional Chinese medicine Dan’e Fukang Decoction can promote blood circulation and remove blood stasis, regulate menstruation and alleviate pain, and relieve Qi stagnancy in the liver [10–11] (gynecological clinical symptoms are always predominately liver diseases. According to TCM theory, liver catharsis disorder leads to Qi disorder, while Chong Ren disorder causes various syndromes, leading to various gynecological diseases. Therefore, gynecological diseases can be treated from the perspective of the liver, so as to receive satisfactory outcomes), which is used for adenomyosis patients with blood stasis caused by irregular menstruation, dysmenorrhea, and menstrual discomfort, with excellent efficacy.

The cause of endometriosis currently remains unclear, which requires in-depth exploration of the exact effect of different approaches. TCM researchers create some effective formulas through the use of basic theory and method characteristics of TCM while referring to Western research, so as to achieve the purpose of alleviating pain, improving ovarian function, regulating immune function, improving uterine status, and preventing recurrence, ultimately improving the conception rate. Therefore, this study may provide more references for investigating the treatment approaches of AM through the comparison of the curative effect of TCM (Dan’e Fukang Decoction) and some common Western medicine regimens.

## Study subjects and methods

### Study subjects

A total of 210 patients with adenomyosis collected by our hospital from January 1, 2022 to July 1, 2023 were selected as the study subjects through convenience sampling and randomly divided into Group A (Dan’e Fukang Decoction), Group B (Dienogest), and Group C (Goserelin + Mirena), with 70 cases in each group. The study was approved by the Ethics Committee of the hospital, and all patients signed informed consent forms. Ethics number: 2021-YJS Lun-004. Approval date: December 12.13,2021.

Inclusion criteria: (1) Patients who meet the clinical diagnostic criteria of the disease: (2) Aged 18–50 years; (3) Patients with obvious menstrual abdominal pain; (4) Patients with a menstrual cycle of 21–35 days; menstrual volume increased than previous volume and changed significantly; (5) Ultrasound showed that the uterus was significantly enlarged; (6) Serum tumor marker CA125 elevated; (7) Gynecological examination showed irregular uterine shape, uneven surface, with or without nodules and masses; (8) Patients who were unable to conceive due to adenomyosis; (9) Patients who could adhere to effective non-oral contraceptive measures during the observation period; (10) Patients who had absolute informed consent to this study for agreeing to use investigational drugs or refusing to take other treatments.

Exclusion criteria: (1) Patients who were allergic to the investigational drugs and had contraindications; (2) Patients who took hormone drugs 3 months before enrollment; (3) Patients with a history of heart disease, liver disease, endocrine or autoimmune diseases; (4) Patients with ovarian function damage; endometrial lesions; severe liver and kidney dysfunction; taking hormone therapy half a year before treatment; (5) Patients with amenorrhea lasting ≥ 3 months; (6) Gynecological examination suggested suspicious malignancy; (7) Patients who were unable to cooperate with the completion of the study.

### Study methods

Patients were randomly allocated to the three treatment groups using a computer-generated randomization sequence stratified by age (< 35 vs. ≥35 years) and baseline uterine volume (≤ 150 cm³ vs. >150 cm³). Allocation concealment was ensured via sealed opaque envelopes opened after enrollment. Baseline characteristics (e.g., age, BMI, symptom severity) were compared across groups to confirm homogeneity, and no significant differences were observed (Table 1), minimizing selection bias.

**Table 1:** Comparison of general data

Item	Group A (*n* = 70)	Group B (*n* = 70)	Group C (*n* = 70)	F/χ^2^ value	*P* value
Age (Y)	35.09 ± 2.48	34.83 ± 3.98	35.81 ± 3.69	1.541	0.217
BMI (kg/m^2^)	22.56 ± 1.10	22.38 ± 1.05	22.26 ± 1.50	1.063	0.347
Gravidity	2.10 ± 0.49	2.10 ± 0.39	2.13 ± 0.51	0.089	0.915
Parity	1.59 ± 0.63	1.61 ± 0.49	1.54 ± 0.81	0.211	0.810
Endometrial thickness (mm)	5.85 ± 1.28	5.91 ± 1.37	6.20 ± 1.19	1.424	0.243
Duration of disease (Y)	3.26 ± 1.45	3.51 ± 1.20	3.27 ± 1.55	0.737	0.480
Clinical symptoms					
Dysmenorrhea symptoms	35	33	36	0.267	0.875
Enlarged uterus	44	32	33	5.074	0.079
Compression symptom	16	15	20	1.088	0.581
Menstrual disorder	11	10	16	2.034	0.362

Group A: Dan’e Fukang Decoction (Yunnan Shengke Pharmaceutical Co., Ltd., GYZ20025253) was used for treatment, which was taken on the 15th day before menstruation for 15 days, 15 g/time, twice a day, and stopped during menstruation, lasting for a total of 6 months.

Group B: Dienogest (Bayer Healthcare Co., Ltd., Approval No.: H20180090) was given, 2 mg/time, once a day, with a fixed duration of treatment for 6 months.

Group C: Goserelin (Approval No.: J20100126, 3.6 mg/vial) was given subcutaneously, 3.6 mg/time, once every 28 days, lasting for a total of 3 injections. The Mirena IUD (Bayer Healthcare Co., Ltd., Guangzhou Branch, Approval No.: GYZJ20140088) was placed by a designated person 28 days after the last injection of Goserelin. Method: After 5–7 days of menstruation, the vagina was dilated under ultrasound guidance. The filaments of the Mirena IUD were pulled apart before slowly inserting the IUD through the cervical opening by pulling the filaments. After ensuring complete contact between the cervix and the positioning block, the filaments were released, with the inserter removed, and then the filaments were cut off, followed by placing the IUD at the fundus about 2 cm away from the cervix. The procedure was completed after confirming the IUD was securely placed.

### Data collection

The general data of patients were collected, including the following: uterine volume, pelvic pain assessed via Visual Analog Scale (VAS), estradiol (E2), follicle-stimulating hormone (FSH), carbohydrate antigen (CA125), and vaginal bleeding volume (PBAC) pre-treatment and at 3 and 6 months post-treatment; adverse reactions during treatment; and follow-up data.

General Data: Age, BMI, gravidity, parities, endometrial thickness, duration of the disease, and symptoms.

Pain Assessment: The severity of pelvic pain and dyspareunia was observed using the Visual Analog Scale (VAS) [12]. The scoring ranges from 0 to 10, where 0 indicates no pain and 10 indicates the most severe pain. Patients marked their pain experience accordingly: 0 points: No pain; 1–3 points: Mild pain; 4–6 points: Moderate pain; 7–9 points: Severe pain; 10 points: Unbearable pain (excruciating pain).

Menstrual Volume: Menstrual blood loss was quantified using the PBAC method [13], with results utilized as assessment indicators. The degree of blood staining on each sanitary pad during menstruation was adopted to evaluate the menstrual volume of patients: the blood-stained area does not exceed one-third of the pad = 1 point; the blood-stained area covers one-third and three-fifths of the pad = points; the blood-stained area almost covers the entire pad = 20 points. Menstrual volume = Total score /100/80m. Meanwhile, small blood clots (< 1 yuan coin size) = 1 point, and larger clots (> 1 yuan coin size) = 5 points. For blood loss that cannot be expressed by clots, it is estimated as a fraction of the recorded volume. The score, quantity, and days of each sanitary pad were recorded in a counting and scoring sheet, with instructions given to subjects to collect menstrual blood using sanitary pads as much as possible to ensure accuracy.

Uterine Changes: Uterine volume was monitored, with particular attention paid to changes in endometrial thickness. Uterine volume can be calculated using the formula (Uterine Volume = 0.52×Long diameter×Anteroposterior diameter×Thickness) after measuring the patient via color Doppler ultrasound [14].

E2, FSH, and CA125 were measured by collecting peripheral venous blood from patients (fasting samples were taken in the morning within 3 days after menstruation ends).

Follow-Up Data: Data on pelvic pain scores (VAS) and changes in uterine volume 12 months post-treatment were collected.

### Statistical analysis

Statistical analysis was performed using IBM SPSS Statistics version 26.0 (IBM Corp., Armonk, NY, USA) [15]. the normality test was performed using the K-S method, and measurement data following normality were expressed as mean ± standard deviation (x ± s). One-way analysis of variance was utilized to compare means among multiple groups, pairwise comparison was conducted using the LSD method, and repeated measurement data were analyzed through repeated measurement analysis of variance. Data not following normal distribution were expressed as M (Q1, Q3), and the Kruskal-Wallis H rank sum test was used for multi-group comparisons. Count data were expressed as frequency (n) or rate (%), and *the χ2* test was used. Bilateral *P* < 0.05 was considered statistically significant, and bilateral *P* < 0.05/N (N for the number of pairwise comparisons) was considered statistically significant in pairwise comparison of count data.

## Results

### General data

The results showed a mean age of 35.09 ± 2.48 years in Group A, with mean gravidity of 2.10 ± 0.49 times and mean parity of 1.59 ± 0.63 times; the mean age of Group B was 34.83 ± 3.98 years, with mean gravidity of 2.10 ± 0.39 times and mean parity of 1.61 ± 0.49 times; and the mean age of Group C was 35.81 ± 3.69 years, with mean gravidity of 2.13 ± 0.51 times and mean parity of 1.54 ± 0.81 times. No significant differences were observed in age, BMI, gravidity, and parity among the 3 groups (*P* > 0.05), as shown in Table 1.

### Comparison of relevant clinical indicators

The effects of different approaches on uterine volume, pelvic pain scores (VAS), and PBAC in AM patients were investigated using the one-way repeated measures analysis of variance, and the Shapiro-Wilk test showed that the data of each group followed an approximately normal distribution (*P* > 0.05). In the meantime, Mauchly’s test of sphericity indicated an equal variance-covariance matrix in each group (*P* > 0.05), and the data were expressed as x ± s, as shown in Table 2, and the results were summarized and described as follows:

**Table 2:** Relevant clinical indicators

Indicator	Time	Group A (*n* = 70)	Group B (*n* = 70)	Group C (*n* = 70)	F _time_ /*P* _time_ value	F _interaction_/*P* _interaction_ value	F _treatment_/*P* _treatment_ value
Uterus Volume	Pre-treatment ^a^	146.08 ± 5.35	146.60 ± 3.39	147.03 ± 2.12	10775.137 /<0.001	1094.561 /<0.001	3588.864 /<0.001
	3 months of treatment ^b^	76.15 ± 3.96	114.08 ± 4.81	133.74 ± 3.47			
	6 months of treatment ^b^	70.05 ± 2.88	106.53 ± 3.67	98.14 ± 4.37			
Pelvic Pain (VAS)	Pre-treatment ^a^	4.74 ± 1.37	4.61 ± 1.21	4.39 ± 1.17	240.624 /<0.001	9.859 /<0.001	25.389 /<0.001
	3 months of treatment ^b^	2.57 ± 0.75	3.40 ± 0.81	3.04 ± 0.89			
	6 months of treatment ^b^	2.11 ± 0.50	3.31 ± 1.19	2.31 ± 0.55			
PBAC	Pre-treatment ^a^	99.20 ± 8.16	100.15 ± 10.63	101.73 ± 14.03	2368.378 /<0.001	136.452 /<0.001	320.101 /<0.001
	3 months of treatment ^b^	68.51 ± 6.83	71.35 ± 9.52	39.43 ± 7.84			
	6 months of treatment ^b^	58.75 ± 10.01	28.46 ± 8.42	20.82 ± 6.85			

Note: VAS: Visual Analogue Scale, PBAC: Menstrual volume, a: Without significant differences between groups, b: With significant differences between groups

The interaction of uterine volume, pelvic pain (VAS), and time*treatment of PBAC was significant among the 3 groups (F _uterine volume_=1094.561, F _pelvic pain (VAS)_ = 9.859, F _PBAC_=136.452, *P* < 0.001 for all), indicating that the separate effect changes in uterine volume, pelvic pain (VAS), and PBAC were different among the 3 groups at the 3 time points. Additionally, the scores of each indicator changed with time in all 3 groups (F _uterine volume_=10775.137, F_pelvic pain (VAS)_ = 240.624, F_PBAC_ = 2368.378, *P* < 0.001). Moreover, different treatments had different effects on uterine volume, pelvic pain (VAS), and PBAC among the 3 groups (F _uterine volume_=3588.864, F _pelvic pain (VAS)_ = 25.389, F _PBAC_=320.101, *P* < 0.001 for all). Further pairwise comparison showed that the uterine volume at 3 and 6 months of treatment was ranked as Group C > Group B > Group A and Group B > Group C > Group A, respectively; pelvic pain VAS score at 3 and 6 months of treatment was ranked as Group B > Group C > Group A and Group B > Group C = Group A, respectively; and PBAC at 3 and 6 months of treatment was ranked as Group B > Group A > Group C and Group A > Group B > Group C, respectively.

### Comparison of serum markers

At the same time, the effects of different approaches on E2, FSH, and CA125 in AM patients were also investigated using the one-way repeated measures analysis of variance, and the Shapiro-Wilk test revealed an approximately normal distribution of data for each group (*P* > 0.05). Besides, Mauchly’s test of sphericity suggested an equal variance covariance matrix in each group (*P* > 0.05), and the data were expressed as x ± s, as shown in Table 3, and the results were summarized and described as follows:

**Table 3:** Serum markers

Marker	Time	Group A (*n* = 70)	Group B (*n* = 70)	Group C (*n* = 70)	F _time_ /*P* _time_ value	F _interaction_/*P* _interaction_ value	F _treatment_/*P* _treatment_ value
	Pre-treatment ^a^	350.01 ± 30.07	342.91 ± 33.88	352.42 ± 33.66	5116.507 /<0.001	3.243 /0.012	5.082 /0.007
	3 months of treatment ^b^	217.33 ± 26.20	200.93 ± 21.02	212.51 ± 29.27			
	6 months of treatment ^b^	104.23 ± 13.02	105.02 ± 11.69	99.94 ± 11.30			
	Pre-treatment ^a^	9.60 ± 1.22	9.34 ± 1.19	9.21 ± 1.20	2752.047 /<0.001	3.377 /0.014	7.375 /0.001
	3 months of treatment ^b^	6.13 ± 0.66	6.53 ± 0.67	5.86 ± 0.72			
	6 months of treatment ^a^	2.97 ± 0.78	3.04 ± 0.69	2.90 ± 0.46			
	Pre-treatment ^a^	125.87 ± 32.42	114.23 ± 22.30	122.51 ± 24.21	1668.382 /<0.001	2.150 /0.105	6.113 /0.003
	3 months of treatment ^b^	40.37 ± 10.89	36.35 ± 9.08	35.85 ± 8.82			
	6 months of treatment ^a^	38.35 ± 6.58	36.76 ± 8.34	36.96 ± 8.93			

Note: E2: Estradiol; FSH: Follicle stimulating hormone; CA125: Carbohydrate antigen 125; a: Without significant differences between groups; b: With significant difference between groups

The interaction of time*treatment of E2 and FSH was significant in the 3 groups (F_E2_=3.243, F_FSH_=3.377, *P* < 0.05 for all), while the interaction of time*treatment of CA125 was not significant (F_CA125_=2.150, *P* > 0.05). Additionally, the scores of each indicator changed with time in all 3 groups (F_E2_=5116.507, F_FSH_=2752.047, F_CA125_=1668.382, *P* < 0.001). Moreover, different treatments had varying effects on E2, FSH, and CA125 levels in the 3 groups (F_E2_=5.082, F_FSH_ =7.375, F_CA125_=6.113, *P* < 0.001 for all). Further pairwise comparison indicated that E2 at 3 and 6 months of treatment was ranked as Group C = Group A > Group B, respectively; FSH at 3 months of treatment was ranked as Group B > Group A > Group C, with no significant differences between groups at 6 months of treatment (*P* > 0.05); and CA125 after 3 months of treatment was ranked as Group B = Group C > Group A, and there were no significant differences between groups at 6 months of treatment (*P* > 0.05).

### Occurrence of adverse reactions

The results revealed that at 3 months of treatment, 15 patients in Group A experienced adverse reactions, with an incidence of 21.43%; 9 patients in Group B had adverse reactions, with an incidence of 12.86%; and 36 patients in Group C suffered adverse reactions, with an incidence of 51.43%. Significant differences were observed in the incidence of adverse reactions among the 3 groups (*χ*^*2*^ = 28.140, *P* < 0.001). Further comparison showed a lower incidence of adverse reactions in Groups A and B than in Group C (*P* < 0.017), as shown in Table 4.

**Table 4:** Adverse reactions

Time	Group	Nausea	Breast tenderness	Headache/ insomnia	Gastrointestinal reactions	IUD displacement	Total	Incidence (%)
3 months	Group A (*n* = 70) ^a^	9	6	0	0	0	15	21.43
	Group B( *n* = 70)^a^	0	2	9	0	0	9	12.86
	Group C (*n* = 70)	6	6	33	15	6	36	51.43
	*χ*^*2*^ value							28.140
	*P* value							< 0.001
6 months	Group A (*n* = 70)	2	4	0	0	0	6	8.57
	Group B (*n* = 70) ^a^	0	1	2	0	0	3	4.29
	Group C(*n* = 70)	1	2	15	5	2	15	21.43
	*χ*^*2*^ value							11.008
	*P* value							0.004

Note: a: Significantly different compared with Group C

**Fig. 1:**
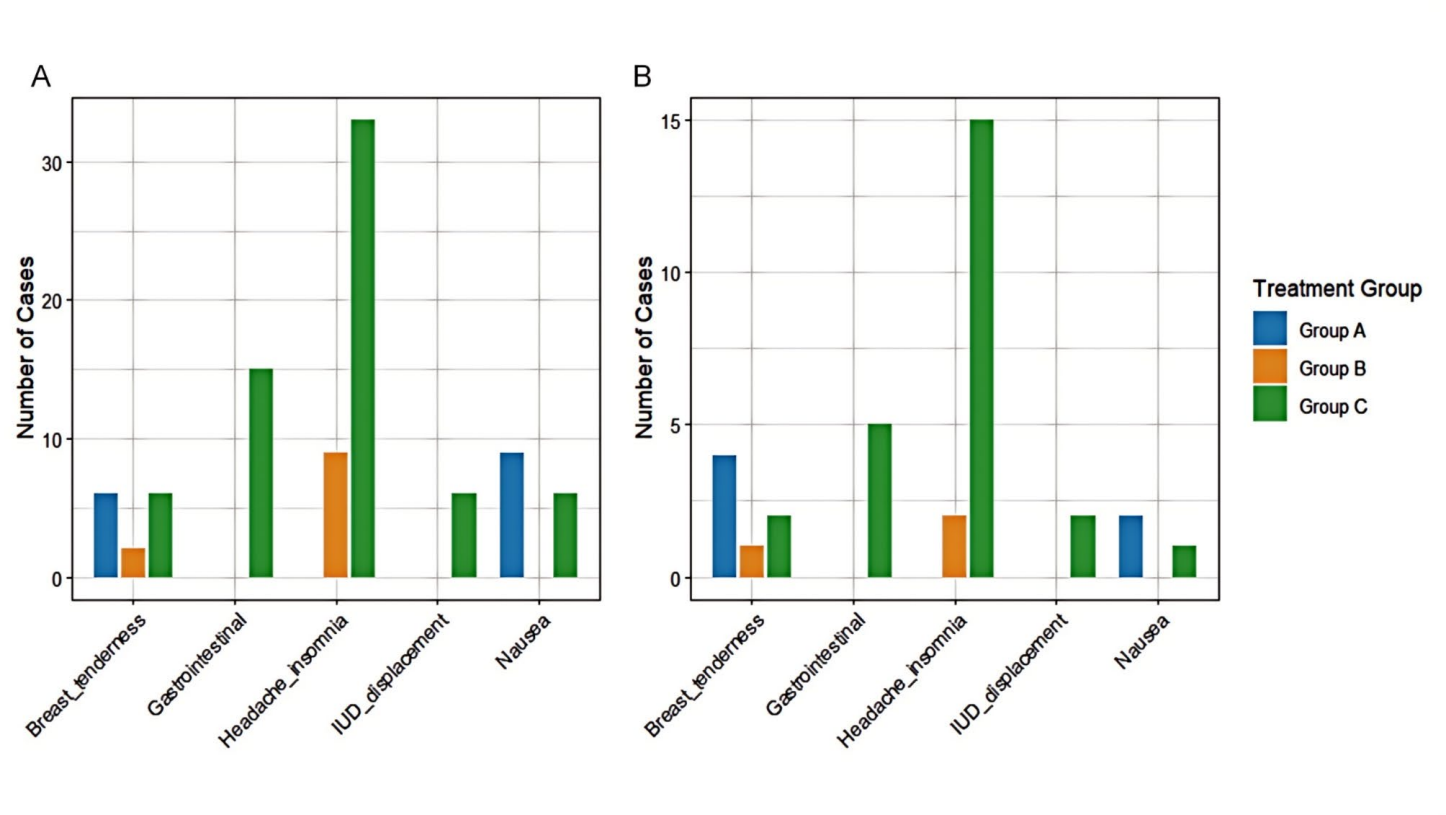
Adverse reactions at 3 and 6 months. Grouping: Group A (Dan’e Fukang Decoction), Group B (Denogestrel), Group C (Goserelin + Manyue Le). Adverse reaction types: Nausea, Breast tenderness, Headache/Insomnia, Gastrointestinal reactions, IUD displacement. The horizontal axis represents the types of adverse reactions, and the vertical axis represents the number of cases. Color differentiation groups: Group A (blue), Group B (orange), Group C (green)

At 6 months of treatment, meanwhile, 6 patients in Group A suffered adverse reactions, with an incidence of 8.57%; 3 patients in Group B experienced adverse reactions, with an incidence of 4.29%; and 15 patients in Group C had adverse reactions, with an incidence of 21.43%. There were significant differences in the incidence of adverse reactions among the 3 groups (*χ*^*2*^ = 11.008, *P* = 0.004). Further comparison showed a lower incidence of adverse reactions in Group B than in Group C, with no significant differences observed between Groups A and B (*P* > 0.017). See Table 4; Fig. 1.

### Uterine volume and pelvic pain (VAS) scores after 12 months of treatment

**Fig. 2:**
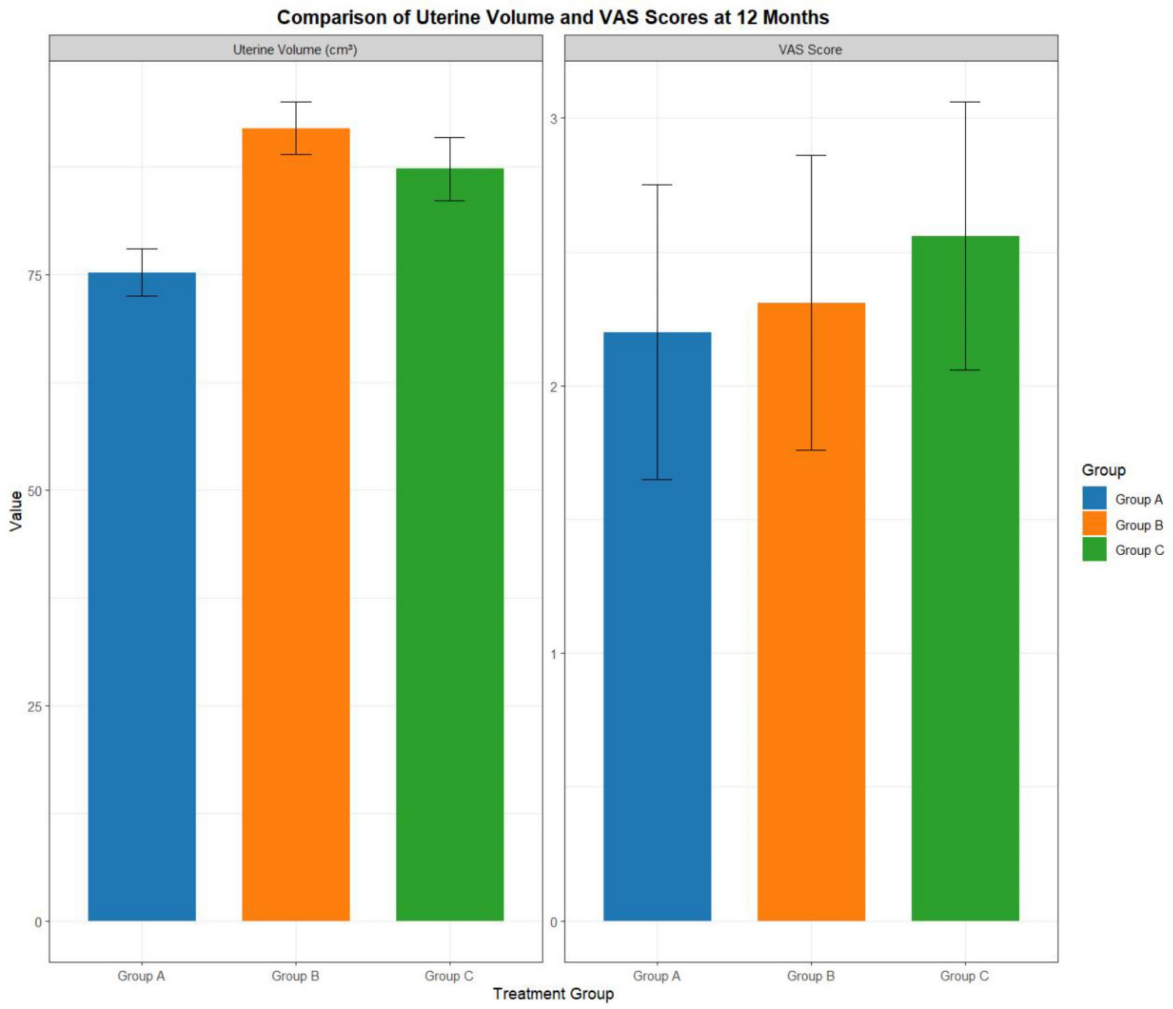
Comparison of uterine volume and VAS scores at 12 months. Grouping: Group A (Dan’e Fukang Decoction), Group B (Denogestrel), Group C (Goserelin + Manyue Le). The horizontal axis represents the treatment group, the vertical axis represents the indicator value, and the error line represents the standard deviation (SD). Color differentiation groups: Group A (blue), Group B (orange), Group C (green)

After 12 months of treatment, significant differences were observed in uterine volume (F = 523.380, *P* < 0.001) and pelvic pain scores (VAS) (F = 8.093, *P* < 0.001) among the 3 groups. Additionally, further comparison revealed that the uterine volume was ranked as Group B > Group C > Group A and the pelvic pain scores (VAS) were ranked as Group C > Group B = Group A. See Table 5; Fig. 2.

**Table 5:** Comparison of uterine volume and pelvic pain scores (VAS) after 12 months of treatment

Item	Group A (*n* = 70)	Group B (*n* = 70)	Group C (*n* = 70)	F value	*P* value
Uterine volume	75.28 ± 2.75	92.04 ± 3.02	87.29 ± 3.64	523.380	< 0.001
Pelvic pain score (VAS)	2.20 ± 0.55	2.31 ± 0.55	2.56 ± 0.50	8.093	< 0.001

Note: VAS: Visual Analogue Scale

## Discussion

Adenomyosis (AM) is a common gynecological disease that usually occurs in women aged 30–50 years with a history of childbearing, and the incidence of symptomatic adenomyosis is around 30-35%, with most patients aged 32–38 years [3]. Women of childbearing age with AM experience a significant decline in the quality of life as they often suffer increasingly severe dysmenorrhea, increased menstrual flow, larger uterine volume, and even infertility, resulting in considerable physical and psychological distress for the patients [4].

Dan’e Fukang Decoction is a pure traditional Chinese medicine compound formulation consisting of Rhizoma Sparganii, Yunnan Bupleurum Chinense, Rhizoma Curcumae, Angelica Sinensis, Radix Paeoniae Rubra, Rhizoma Corydalis, Licorice, Cyperus Rotundus, Panax Notoginseng, and Salvia Miltiorrhiza Bunge. The formulation includes Bupleurum Chinense and Angelica Sinensis, which have similar central inhibitory effects and promote uterine contractions. It can shorten bleeding and coagulation times, with anti-platelet aggregation and thrombolytic effects, as well as analgesic and anti-inflammatory properties. The use of Dan’e Fukang Decoction has demonstrated significant improvement in dysmenorrhea of mild, moderate, and severe severity [16]. A prospective RCT study included 172 patients with adenomyosis who were randomly assigned to the treatment group and the control group receiving gestrinone. The treatment group was given 15 g of Dan’e Fukang Decoction twice daily until menstruation ended, for a total of 3 menstrual cycles, while the control group took subcutaneous injection of leuprorelin starting from the 1st to 5th day of the menstrual cycle. The primary observation indicators included efficacy, symptom improvement, and safety. The results showed that the combination of Dan’e Fukang decoction and subcutaneous injection of leuprorelin can not only improve symptoms such as dysmenorrhea, heavy menstrual flow, abdominal pain, and uterine enlargement in patients with adenomyosis, but also reduce the recurrence rate [17]. For the most common symptoms, such as dark menstrual blood with clots, breast tenderness before or during menstruation, and emotional disturbances, treatment aimed at regulating qi and invigorating blood circulation is required, and Dan’e is a representative traditional Chinese medicine for this purpose, which can effectively alleviate primary and secondary dysmenorrhea and improve symptoms like dark menstrual clots, breast swelling, and breast pain. Additionally, it has no adverse effects or toxic side effects on essential tissues and organs [18–19].

Goserelin, by contrast, is a synthetic decapeptide compound and a gonadotropin-releasing hormone agonist commonly used to treat endometrial atypical hyperplasia. Lv et al. [20] have found that goserelin has a significant inhibitory effect on the hypothalamic-pituitary-gonadal axis, effectively reducing estrogen secretion, which helps correct excessive proliferation of the endometrial glands, improves endometrial thickness, and reduces uterine volume. However, the effectiveness of this drug used alone is limited, and long-term use may lead to numerous adverse reactions, with a high probability of recurrence upon discontinuation [21]. In recent years, the intrauterine placement of the Mirena IUD has demonstrated satisfactory results in treating conditions such as increased menstrual volume, endometriosis, and adenomyosis. Additionally, the research by Li Qing et al. [22] concluded that the placement of the Mirena IUD is a relatively safe and effective treatment option for patients wishing to preserve fertility. Specifically, Mirena is a “T”-shaped contraceptive stent containing 52 mg levonorgestrel hormone, which releases a constant daily dose of 20 μg levonorgestrel after intrauterine placement, and the local high concentration of levonorgestrel induces endometrial atrophy, inhibits the proliferation of endothelial cells, and regulates the coagulation function of the endometrium, thereby alleviating dysmenorrhea and reducing menstrual volume [23]. Moreover, it can directly act on the lesions, significantly inhibiting their activity and ultimately relieving the condition of patients [24].

Dienogest, as a novel progestin, has demonstrated strong efficacy, and many experts have verified its effectiveness in alleviating pain in patients with adenomyosis. It has been shown [25] that dienogest can reduce the expression of nerve growth factor (NGF) and the density of nerve fibers associated with adenomyosis, as well as inhibit the secretion of inflammatory factor IL-8 by endometriotic stromal cells [26]. Meanwhile, dienogest at 2 mg/d is effective not only for adenomyosis-related dysmenorrhea but also for pelvic pain and dyspareunia symptoms that are poorly controlled by traditional drugs. In this regard, dienogest effectively prevents adenomyosis symptoms while exhibiting satisfactory tolerance and safety [27], gradually making it one of the optimal drugs for treating dysmenorrhea in patients with adenomyosis.

In this study, the efficacy of the above 3 approaches was compared, and the results showed that goserelin + Mirena and dienogest, were superior to Dan’e Fukang Decoction in terms of reducing uterine volume; Dan’e Fukang Decoction and goserelin + Mirena were superior to dienogest for pain relief; and goserelin + Mirena was more effective than both Dan’e Fukang Decoction and dienogest in reducing bleeding, which is consistent with relevant studies [28]. At the same time, all 3 approaches effectively lowered E2, FSH, and CA125 levels in the patients’ blood and were ranked as dienogest > Dan’e Fukang Decoction > goserelin + Mirena for reducing adverse reactions. In terms of long-term efficacy, goserelin + Mirena was most effective in reducing uterine volume, while Dan’e Fukang Decoction and dienogest demonstrated optimal pain relief. However, given the currently limited research exploring the clinical effects of the 3 approaches, as well as a lack of research evidence for comparison, further investigation is needed.

Existing literature indicates that conservative treatment and surgical intervention each have their advantages and disadvantages in the management of uterine adenomyosis. In terms of short-term efficacy, conservative treatment can effectively alleviate pain and reduce menstrual bleeding, with a symptomatic improvement rate comparable to that of lesion excision, while avoiding surgical trauma and hospitalization [29]. However, regarding long‐term outcomes, conservative treatment is associated with a higher recurrence rate, whereas hysterectomy can provide a definitive cure, albeit at the cost of permanent loss of fertility [30]. For patients wishing to preserve fertility, conservative surgical procedures (such as adenomyomectomy) exhibit a high recurrence rate and an increased risk of postoperative adhesions [31]. Notably, our study found that the combination of Dan’e Fukan decoction with Western medicine maintained reduced uterine volume and low pain scores at 12 months, suggesting its potential as a non‐surgical alternative in select patient populations. Overall, conservative treatment is more appropriate for patients with milder symptoms or those requiring fertility preservation, while surgical intervention is indicated for severe cases or when medical therapy fails, with a careful consideration of the long‐term recurrence risk versus the benefits of functional preservation.

Notably, this study also comes with limitations: (1) All the enrolled patients were from only one hospital, with regional and participant constraints. Additionally, there were issues such as a small sample size, a short study duration, and limited participants, making it necessary to increase the sample size and scope and extend the study duration for more in-depth observation in the future, current study evaluated outcomes up to 12 months post-treatment, demonstrating sustained reductions in uterine volume and pain scores. However, recurrence rates beyond this timeframe remain unexplored. Given the chronic nature of adenomyosis, long-term follow-up studies (e.g., 3–5 years) are warranted to assess recurrence risks and the durability of therapeutic effects; (2) Currently, there is little research comparing the clinical effects of the 3 approaches, along with a lack of research evidence for comparison. Therefore, further exploration is needed. (3) This study utilized a convenience sample of 210 patients without prior sample size calculation, which may limit the generalizability of the findings. Although the groups showed balanced baseline characteristics, the lack of power analysis increases the risk of type II errors. Future research should incorporate formal sample size calculations based on primary outcome effect sizes to ensure adequate statistical power.

## Conclusion

In conclusion, Dan’e Fukang Decoction can effectively relieve pain without changing the normal menstrual physiology, goserelin + Mirena is significantly effective in reducing bleeding and shrinking the uterus, and all 3 approaches demonstrate satisfactory efficacy in reducing E2, FSH, and CA125 levels in the blood of patients. However, Dan’e Fukang Decoction and dienogest demonstrate optimal pain relief with regard to long-term efficacy.

## Data Availability

All data generated or analysed during this study are included in this article. Further enquiries can be directed to the corresponding author.
